# Mosquitoes (Diptera: Culicidae) in Mauritania: a review of their biodiversity, distribution and medical importance

**DOI:** 10.1186/s13071-017-1978-y

**Published:** 2017-01-19

**Authors:** Aichetou Mint Mohamed Lemine, Mohamed Aly Ould Lemrabott, Moina Hasni Ebou, Khadijetou Mint Lekweiry, Mohamed Salem Ould Ahmedou Salem, Khyarhoum Ould Brahim, Mohamed Ouldabdallahi Moukah, Issa Nabiyoullahi Ould Bouraya, Cecile Brengues, Jean-François Trape, Leonardo Basco, Hervé Bogreau, Frédéric Simard, Ousmane Faye, Ali Ould Mohamed Salem Boukhary

**Affiliations:** 1 Unité de recherche Génomes et Milieux, Jeune Equipe Associée à l’IRD (RI3M), Université de Nouakchott Al-Aasriya, Faculté des Sciences et Techniques, Nouveau Campus Universitaire, Nouakchott, BP 5026 Mauritania; 2 Laboratoire d’Ecologie Vectorielle et Parasitaire, Faculté des Sciences et Techniques, Université Cheikh Anta Diop, Dakar, Senegal; 3 Institut Supérieur des Etudes technologiques, Rosso, Mauritania; 4 Maladies Infectieuses et Vecteurs: Ecologie, Génétique, Evolution et Contrôle (MIVEGEC), Unité Mixte de Recherche IRD224-CNRS5290-Université de Montpellier, Institut de Recherche pour le Développement (IRD), Montpellier, France; 5 Unité de Recherche sur les Maladies Infectieuses et Tropicales Emergentes (URMITE), UM 63, CNRS 7278, IRD 198, Inserm 1095, Faculté de Médecine La Timone, Aix-Marseille Université, Marseille, 13385 France; 6 Unité Parasitologie et Entomologie, Département des Maladies Infectieuses, Institut de Recherche Biomédicale des Armées, Marseille, France

**Keywords:** Culicidae, Biodiversity, Mosquitoes, Vector, Rift Valley fever, Dengue, Malaria, Mauritania

## Abstract

Although mosquitoes (Diptera: Culicidae) are important disease vectors, information on their biodiversity in Mauritania is scarce and very dispersed in the literature. Data from the scientific literature gathered in the country from 1948 to 2016 were collected and analyzed. Overall 51 culicid species comprising 17 *Anopheles* spp., 14 *Aedes* spp., 18 *Culex* spp. and two *Mansonia* spp. have been described in Mauritania among which *Anopheles arabiensis*, *Aedes vexans, Culex poicilipes* and *Culex antennatus* are of epidemiological significance. *Anopheles arabiensis* is widely distributed throughout the country and its geographic distribution has increased northwards in recent years, shifting its northern limit form 17°32′N in the 1960s to 18°47′N today. Its presence in the central region of Tagant highlights the great ecological plasticity of the species. Conversely, the distribution of *Anopheles gambiae* (*s.s.*) and *Anopheles melas* has shrunk compared to that of the 1960s. *Anopheles rhodesiensis* and *An. d’thali* are mainly confined in the mountainous areas (alt. 200–700 m), whereas *Anopheles pharoensis* is widely distributed in the Senegal River basin. *Culex poicilipes* and *Cx. antenattus* were naturally found infected with Rift valley fever virus in central and northern Mauritania following the Rift valley outbreaks of 1998 and 2012. Recently, *Ae. aegypti* emerged in Nouakchott and is probably responsible for dengue fever episodes of 2015. This paper provides a concise and up-to-date overview of the existing literature on mosquito species known to occur in Mauritania and highlights areas where future studies should fill a gap in knowledge about vector biodiversity. It aims to help ongoing and future research on mosquitoes particularly in the field of medical entomology to inform evidence-based decision-making for vector control and management strategies.

## Background

Mosquitoes (Diptera: Culicidae) are considered one of the most relevant groups of arthropods in public health [1, 2]. Those belonging to the genera *Aedes*, *Anopheles* and *Culex* are of interest because of their role in the transmission of a variety of human and animal diseases such as Rift Valley fever (RVF), dengue fever (DF), yellow fever (YF), Zika, chikungunya and malaria. RVF, DF and YF are acute febrile mosquito-borne viral diseases of man and animals (RVF) which cause clinical syndromes ranging from an uncomplicated form with fever to hemorrhagic disease in humans and abortions and mortality during epizootics in livestock [3]. Malaria, the deadliest vector-borne parasitic disease worldwide, is caused by a protozoan belonging to the genus *Plasmodium*. According to the World Health Organization (WHO), vector-borne diseases account for 17% of the estimated global burden of all infectious diseases mostly due to malaria and DF [4].

Mosquitoes are dipterans of the suborder Nematocera, all placed within the family Culicidae. Approximately 3500 species and subspecies in 44 genera are recognized globally [5]. The cosmopolitan genera *Anopheles* with seven subgenera involving 460 recognized species, *Culex* with 26 subgenera comprising 763 species and the Old World and Nearctic genus *Aedes* with 70 subgenera including 927 species are the highest in species diversity and most important for public health in the family [5, 6].

Historically, five large outbreaks of RVF occurred in Mauritania in 1987, 1998, 2003, 2010 and 2012 resulting in a high number of human fatalities and major losses in the livestock population [7–12]. Furthermore, the WHO outbreaks and emergencies bulletin reported DF and RVF events in Mauritania in 2015 [13]. Although in this bulletin, the areas in which these outbreaks have occurred were not specified, data from the Mauritanian Ministry of Health have cited Nouakchott, the capital city and the southern Brakna region for DF and RVF, respectively (Ouldabdallahi Moukah, pers. comm*.*).

Moreover, malaria is endemic in the southern regions of the country and in parts of the Saharan region, including Nouakchott, the capital city, where peak transmission occurs in September and October during and shortly after the rainy season [14–17]. Approximately, two-thirds of the resident population of Mauritania is exposed to the risk of malaria.

In Mauritania, knowledge about the Culicidae fauna has been closely related to studies on human health, like the malariometric surveys of 1942 during the colonial period [18] and those of 1960s to assess malaria epidemiology [19–21] and more recently after the recurrent RVF outbreaks [9, 12, 22, 23]. It is worth noting that there are no published data on mosquitoes in the country for the period 1970–1990 probably because of the prolonged period of drought in the 1970s and 1980s in the Sahel [24] during which food security had become a priority over public health in most Sahelian countries.

Although mosquitoes are important disease vectors, information on mosquito biodiversity in Mauritania is scarce and notably dispersed in the literature. Therefore, there is an urgent need for more detailed understanding of the biodiversity, distribution and ecology of mosquito species known to occur in the country. This paper provides the first comprehensive review on mosquitoes in Mauritania and analyses data collected from the scientific literature and published reports available within the country.

### Salient data on Mauritania

**Fig. 1:**
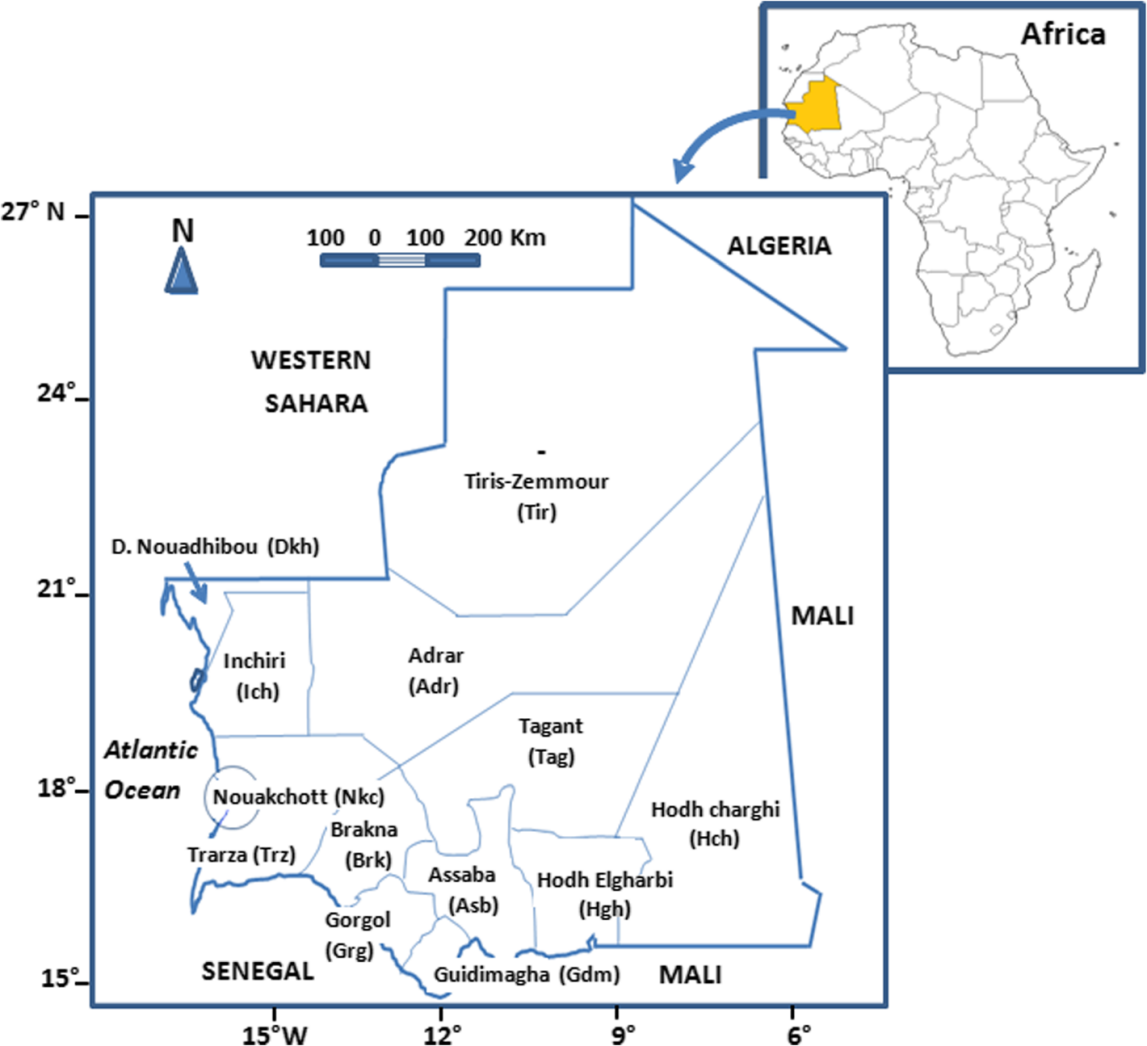
Map of Mauritania with its administrative regions. Administrative district codes are provided in parentheses

Mauritania (15°–27° N latitude and 5°–17° W longitude) is in northwest Africa at the intersection of the Maghreb region and sub-Saharan West Africa (Fig. 1). It covers an area of 1,030,700 km^2^, and has a population of 3,378,250, and a mean population density of 3.3 persons/km^2^. The population is predominantly distributed (i.e. 80% of inhabitants) in the southern Sahelian region and along the Senegal River. Settlement of nomad populations and rural exodus, partly related to the periods of drought in the 1970s, 1980s and 1990s, are the most significant demographic phenomena that have occurred in Mauritania since the country’s independence in 1960. Whereas the proportion of urban population was 9% in 1965, it increased to 22.7, 46.7 and 60% in 1977, 2005 and 2010, respectively. Over the same period, the nomad population rapidly decreased from 65% in 1965 to 12, 6 and 2% in 1988, 2000 and 2013, respectively [25]. Mauritania has a large livestock population with 1,247,000 camels, 1,657,000 cattle, 12,555,000 small ruminants (sheep and goats) and an important population of equines (donkeys and horses) estimated at 212,000 [26]. The livestock density is higher in southern Mauritania compared to that of its arid northern part.

Despite being largely arid, there are several temporary and permanent wetland areas in Mauritania. The most important are the Senegal River Delta, the protected coastal Banc d’Arguin National Park and the Diawling National Park, the natural lakes of Aleg, Mal, Rkiz, mare de Kankoussa, mare de Mahmoude and more than 300 oases [27, 28].

Mauritania is an arid country with two-thirds of its surface area lying within the arid zone of the Saharan desert (0–100 mm annual rainfall) and the remainder belongs to the Sahelian zone (100–500 mm annual rainfall).

**Fig. 2:**
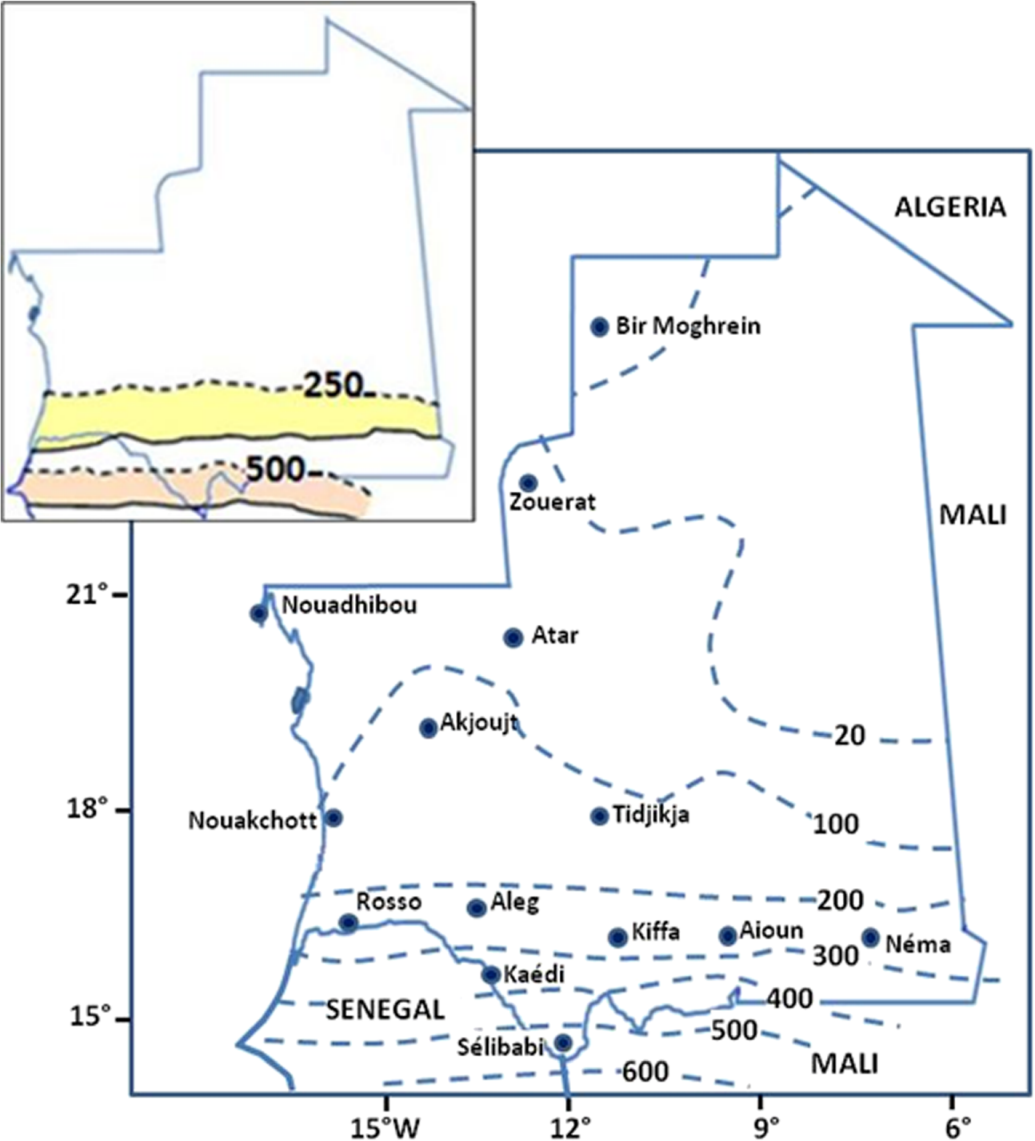
Rainfall isohyets for 1940–1999 in Mauritania. Insert: 250 and 500 mm isohyetal line shifts for the humid period of 1940–1969 (*dotted lines*) and the dry period of 1970–1999 (*continuous lines*). (Adapted from Mahé et al. [30])

Rain is relatively scarce and irregular over the country. It falls between July and September, with an increased gradient from North to South, ranging from less than 50 mm annually in the northern Saharan zone to 500 mm in parts of the southernmost region of Guidimagha (Fig. 2). Studies on the variability of rainfall in Mauritania showed a significant decreasing trend in annual rainfall because of prolonged drought of 1970–1990. For instance, in the Sahelian southern part of the country, average rainfall decreased by 120 mm (39%) between the periods 1933–1969 (310 mm) and 1970–1999 (190 mm) [29] which resulted in a southward movement of 250 and 500 mm rainfall isohyetal lines as reported by Mahé et al. [30] (Fig. 2). However, it is not yet clear whether the unusual heavy rainfalls that occurred in the last decade, particularly in 2003, 2006, 2010 and 2013, are signs of the establishment of sustainable wetter conditions as suggested by De Longueville et al. [31].

Mean annual temperatures over the country range from 20 °C in January to 33.7 °C in June. According to McSweeney et al. [32], the mean annual temperature in Mauritania has increased by 0.9 °C since 1960, with an average rate of 0.19 °C per decade.

### Culicidae fauna in Mauritania

**Fig. 3:**
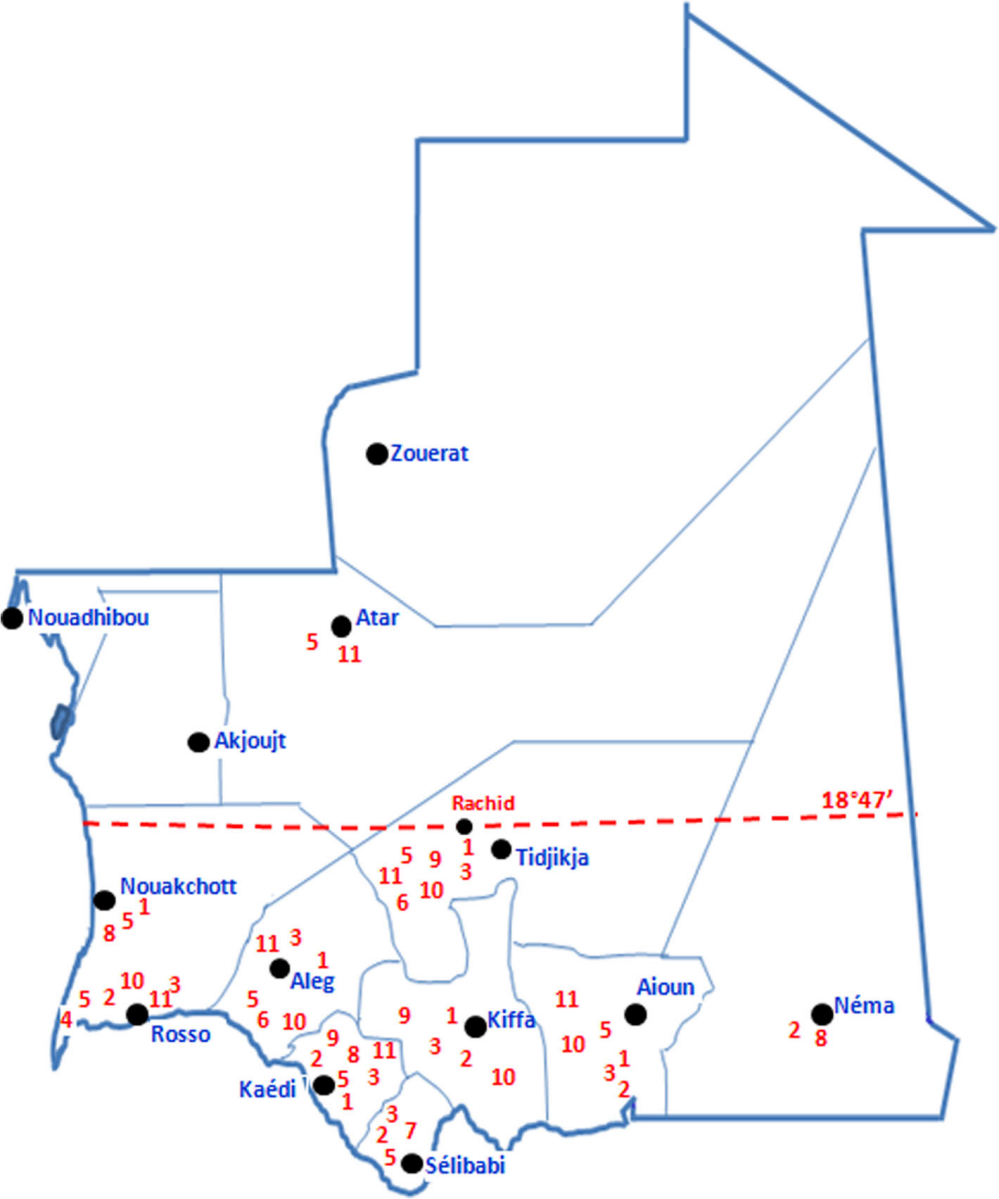
Distribution of mosquitoes of medical importance in Mauritania with indication of the northern limit of *An. arabiensis* (*dotted line*). 1, *An arabiensis*; 2, *An. gambiae* (*s.s.*); 3, *An. funestus*; 4, *An. melas*; 5, *An. pharoensis*; 6, *An. ziemanni*; 7, *An. wellcomei*; 8, *Aedes aegypti*; 9, *Ae. vexans*; 10, *Culex poicilipes*; 11, *Cx. antenattus*

Overall, 51 mosquito species belonging to four genera were described in Mauritania between 1948 and 2016 (Table 1). Published literature before 1970 mentioned 41 mosquito species comprising 13 *Anopheles* spp., 9 *Aedes* spp., 17 *Culex* spp. and 2 *Mansonia* spp., while from 2000 until now 34 mosquito species, involving 15 *Anopheles* spp., 8 *Aedes* spp., 9 *Culex* spp. and 2 *Mansonia* spp., were described. The distribution of mosquitoes of medical importance in Mauritania is given in Fig. 3.

**Table 1:** List of the Culicidae species collected as adult (A) and larva (L), and their distribution in Mauritania

Genus	Species (development stage)		Distribution^a^	References
	Before 1970	Recent (2000-present)		
*Anopheles* spp*.*	*An. arabiensis* (A, L)	*An. arabiensis* (A, L)	Brk, Hch, Hgh, Gmg, Nkc, Tag, Trz	[8, 20, 22, 42, 43, 47, 48]
	*An. coustani* (A, L)	*An. coustani* (A)	Asb, Brk, Grg, Hgh, Trz	[8, 20, 35]
	*An. demeilloni* (A, L)		Hgh	[20]
	*An. d’thali* (A, L)	*An. d’thali* (A, L)	Adr, Hgh, Tag	[19, 20, 34, 44]
		*An. domicola* (A)	Gdm, Grg	[48]
		*An. flavicosta*	na	[40]
		*An. freetownensis* (A)	Hgh	[8]
	*An. funestus* (A, L)	*An. funestus* (A	Asb, Brk, Grg, Gmg; Hgh, Tag, Trz	[8, 9, 18, 20, 22, 23, 35, 47]
	*An. gambiae* (A, L)	*An. gambiae* (A, L)	Asb, Brk, Grg, Hch, Hgh, Trz	[9, 18, 22, 35, 36, 42, 43, 47, 48]
	*An. melas* (A)		Trz	[20, 33]
	*An. pharoensis* (A, L)	*An. pharoensis* (A, L)	Adr, Asb, Brk, Grg, Hgh, Trz, Tag	[8, 9, 18, 20, 22, 23, 35, 41–44, 47, 48]
	*An. pretoriensis* (L)	*An. pretoriensis* (A	Hgh	[8, 12, 20]
	*An. rhodesiensis* (A, L)	*An. rhodesiensis* (A, L)	Adr, Asb, Hgh, Tag	[8, 19, 20, 23, 41–43, 48]
	*An. rufipes* (A, L)	*An. rufipes* (A, L)	Asb, Brk, Grg, Hgh, Tag, Trz	[8, 9, 18, 20, 23, 35, 41–43, 47, 48]
	*An. squamosus* (A, L)	*An. squamosus* (A)	Adr, Asb, Brk, Grg, Hgh, Trz	[8, 9, 20]
		*An. wellcomei* (A)	na	[9]
	*An. ziemanni* (A, L)	*An. ziemanni* (A	Brk, Hgh, Tag	[8, 9, 20, 23, 42, 48]
*Aedes* spp*.*	*Ae. aegypti* (A, L)	*Ae. aegypti* (A, L)	Hch, Gmg, Grg, Nkc	[35, 51, 54, 55]
		*Ae. caspius* (A, L)	Nkc	[51]
	*Ae. vexans* (A)	*Ae. vexans* (A, L)	Adr, Asb, Grg, Hgh, Tag, Trz	[8–10, 21, 23, 43, 57]
		*Ae. dalzieli* (A)	Hgh	[8]
		*Ae. fowleri* (A)	Hgh	[8]
		*Ae. minutus* (A)	Hgh	[8]
		*Ae. sudanensis* (A)	Tag	[23]
	*Ae. vittatus* (A, L)		Gmg, Grg, Hgh	[35, 54, 55]
	*Ae. luteocephalus* (A)		Grg	[21]
	*Ae. scatophagoides* (A)		Grg, Gmg	[21, 35]
	*Ae. metallicus* (A)		Grg	[21]
	*Ae. ochraceus* (A)	*Ae. ochraceus* (A)	Grg, Hgh	[8, 9, 21]
	*Ae. hirsutus* (A)		Hgh	[35]
	*Ae. argenteopunctatus* (A)		Hch	[35]
*Culex* spp*.*	*Cx. antennatus* (A, L)	*Cx. antennatus* (A)	Adr, Brk, Grg, Hgh, Tag, Trz	[8, 9, 21, 23, 35, 41]
	*Cx. poicilipes* (A, L)	*Cx. poicilipes* (A)	Asb, Brk, Grg, Hgh, Tag, Trz	[8, 9, 21, 23, 35, 41]
	*Cx. ethiopicus* (A, L)	*Cx. ethiopicus* (A)	Asb, Brk, Grg, Hgh, Tag	[8, 9, 21, 35]
		*Cx. quinquefasciatus* (A)	Dkh, Ich, Tir, Trz, Tag,	[8, 23, 41, 43]
	*Cx. duttoni* (L)		Gmg, Hgh	[35]
	*Cx. grahami* (L)		Brk, Hgh	[35]
	*Cx. annulioris* (L)		Hgh	[35]
	*Cx. univittatus* (A, L)		Adr, Asb, Brk, Grg, Hgh, Tag, Trz	[21, 35]
	*Cx. simpsoni* (L)		Grg, Hgh	[21, 35]
	*Cx. pipiens* (A, L)		Adr, Hgh	[21, 35]
	*Cx. nebulosus* (A, L)		Gmg	[35]
	*Cx. fatigans* (A, L)		Grg, Nkc, Trz	[21, 35]
	*Cx. decens* (A, L)	*Cx. decens* (A)	Adr, Asb, Hgh, Tag,	[8, 21, 23, 35, 41]
	*Cx. perfuscus* (A)	*Cx. perfuscus* (A)	Adr, Asb, Hgh	[8, 21, 35, 41]
	*Cx. neavei* (A)	*Cx. neavei* (A)	Adr, Hgh, Tag	[8, 9, 23, 35]
	*Cx. perexiguus* (A, L)		Adr, Asb, Hgh, Grg, Nkc, Tag, Trz	[21]
	*Cx. tritaeniorhynchus* (L)	*Cx. tritaeniorhynchus* (A)	Adr, Brk, Trz	[9, 35]
	*Cx. tigripes* (A, L)	*Cx. tigripes* (A)	Asb, Grg, Hgh, Trz	[21, 35]
*Mansonia* spp*.*	*Ma. uniformis* (A)	*Ma. uniformis* (A)	Adr, Brk, Grg, Hgh, Tag, Trz	[9, 10, 21, 23, 35]
	*Ma. africana* (A)	*Ma. africana* (A)	Gmg, Grg	[9, 21, 35]

*Abbreviations*: *Adr* Adrar, *Asb* Assaba, *Brk* Brakna, *Dkh* Dakhlet Nouadhibou, *Gdm* Guidimagha, *Grg* Gorgol, *Hch* Hodh Charghi, *Hgh* Hodh Elgharbi, *Ich* Inchiri, *Nkc* Nouakchott, *Tag* Tagant, *Tir* Tiris Zemmour, *Trz* Trarza, *na* not avaliable

^a^District codes are as in Fig. 1

### *Anopheles* spp.

Until 1960, only six species of *Anopheles* were known to occur in Mauritania namely *An. gambiae*, *An. melas*, *An. funestus*, *An. pharoensis*, *An. rufipes* and *An. d’thali* [18, 33, 34]. In a malariometric survey conducted during the dry season extending from December 1962 to April 1963, Maffi [19] searched for *Anopheles* larvae in a limited number of breeding sites in southern Mauritania (Tagant, Brakna, Gorgol, Trarza, Assaba and Hodh Elgharbi). The author confirmed the presence of *An. gambiae*, *An. pharoensis*, *An. d’thali* and *An. rufipes* larvae in the breeding sites and showed the presence of *An. rhodesiensis*.

This work was followed by a more comprehensive work by Hamon et al. [20, 21]. These authors conducted extensive surveys to assess malaria endemicity in Adrar, Assaba, Trarza, Gorgol and Hodh Elgharbi regions (Guidimagha, the wettest region of Mauritania had not been prospected) during the dry season between November 1962 and March 1963 and shortly after the wet season (October and November) in 1963. Their investigations were not only focused on *Anopheles* mosquitoes but also considered other arthropods of medical importance. They reported the presence of the following 12 *Anopheles* species and subspecies in the prospected zones: *An. funestus*, *An. gambiae* (*sensu lato*) (*s.l.*), *An. pharoensis*, *An. rufipes*, *An. melas*, *An. d’thali*, *An. rhodesiensis*, *An. coustani*, *An. ziemmani*, *An. pretoriensis*, *An. squamosus* and *An. demeilloni*. Among these, *An. gambiae* (*s.l.*) and *An. funestus* are the major malaria vectors in Africa. Entomological survey conducted by Pichon & Ouedraogo [35] aiming at the search of areas fulfilling the conditions for the emergence of yellow fever epidemics confirmed the findings of Hamon et al. [20, 21]. Coz [36] in his work conducted in the late of 1960s in southeastern Mauritania, reported the presence of only *An. gambiae* (species A), later known as *Anopheles gambiae* (*s.s.*) in five sites, including the very dry area of Boutilimit (17°32′N) belonging to the Trarza region. The author considered the presence of *An. gambiae* (*s.s.*) in the very dry areas of Mauritania difficult to explain but conjectured that relict populations of this species may have remained after wider distribution of this species before desertification.

During the survey conducted in 1984 by Baudon et al. [37] at the Foum gleita Dam in the Gorgol region during the dry season, no anopheline was caught, although in the same period, specimens of *An. gambiae* (*s.s.*), *An. arabiensis* and *An. melas*, were captured in several Senegalese localities on the other side of the Senegal river valley [38].

In 1996 and 1997, Molez & Faye [39] studied a south-north transect from Boghé on the Senegal River (middle Delta) to Rachid (18°47′N), in the stony desert plateau of Tagant region. They noted the similitude in the composition of the mosquito fauna between northern Senegal and southern Mauritania along the Senegal River with *An. arabiensis* found throughout the transect and *An. rhodesiensis* captured only in the Tagant region. *Anopheles flavicosta* was also reported in Mauritania, but the location where it was found was not specified [40]. More recently, Nabeth et al. [41] collected *An. pharoensis*, *An. rhodesiensis* and *An. rufipes* using Centers for Disease Control (CDC)-light traps, from five localities in the Hodh Elgharbi southeastern region of Mauritania following the RVF outbreak of 1998. *Anopheles rhodesiensis* was also reported in two localities of the northern Adrar and the central Tagant regions of Mauritania [42, 43]. However, Trape [44] reported only *An. d’thali* in 8 of 13 localities prospected in the Adrar region and *An. pharoensis*, *An. d’thali* and *An. arabiensis* in the Tagant region during the dry season of 2004. During the RVF outbreak of 2003, an entomological survey covering Trarza, Brakna, Assaba, Tagant and Hodh Elgharbi regions was conducted in October and November of the same year [22]. Among 647 anopheline specimens collected using pyrethrum space-spray catch (PSC) method, *An. gambiae* M form, now known as *An. coluzzii* [45] and *An. arabiensis*, both members of the *An. gambiae* (*s.l.*) complex, represented 92% of the specimens, followed by *An. pharoensis* (5%) and *An. funestus* (3%). Specimens of *An. funestus* were observed only in Brakna region adjacent to the Senegal River basin. In all prospected areas *An. gambiae* (*s.l.*) showed no significant difference in their anthropophilic rates and all *An. funestus* and *An. pharoensis* specimens had fed on human and ovine hosts, respectively. Furthermore, of 394 females of the *An. gambiae* complex tested in Assaba, one *An. arabiensis* was positive for *P. falciparum* circumsporozoite antigen, giving an infection rate of 0.25% in this region and 0.17% in the whole study area. During the same RVF outbreak of 2003, Faye et al. [9] further reported the presence of *An. rufipes*, *An. wellcomei*, *An. ziemanni* and *An. squamosus*, in Trarza, Brakna and Assaba southern regions but the authors did not describe the localities where the mosquitoes were captured since their investigations were focused on the potential vectors of RVF. It is worth noting that in an entomological survey conducted following the RVF outbreak of 2012 five anopheline species including *An. funestus*, *An. pharoensis*, *An. rhodesiensis*, *An. rufipes* and *An. ziemanni* were captured in Moudjeria and Tidjikja in the central region of Tagant, and Tamcheket in the southeastern region of Hodh Elgharbi [23].

Using the PSC method to collect indoor resting mosquitoes in Nouakchott (Saharan zone) and Hodh Elgharbi (Sahelian zone), Mint Lekweiry et al. [46, 47] reported that *An. arabiensis* is the sole anopheline species in Nouakchott and showed the infection of three of 186 specimens (1.6%) with *P. vivax* circumsporozoite proteins using enzyme-linked immunosorbent assay (ELISA), providing the first evidence that this species is likely to play an important role in malaria transmission in the city. They also demonstrated the presence of *An. gambiae* (*s.s.*), *An. funestus*, *An. rufipes*, *An. pharoensis* and *An. arabiensis* in Hodh Elgharbi region but were not able to incriminate any of them in malaria transmission in this region due to negative ELISA results although Hamon et al. [20] found *Plasmodium* sporozoites in the salivary glands of two of 18 female *An. gambiae* collected in Hodh Elgharbi during October and November 1963 and reported an overall sporozoite index of 0.45% (2/444) for all study sites. Similar infection rate was reported by Pichon & Ouedraogo [35] in *An. gambiae* specimens collected from different sites in southern Mauritania.

Specimens of *An. domicola* were recently reported by Ouldabdallahi Moukah et al. [48] in Boghé (Brakna region) and Gouraye (Guidimagha region) located on the Senegal River. This species was already reported in Barkedji and Kédougou in Senegal [49].

Environmental factors associated with anopheline larval habitats and their types have not been extensively studied in Mauritania. The available data were obtained from the works of Maffi [19], Hamon et al. [20] and Ould Ahmedou Salem et al. [50]. Larvae of *An. rhodesiensis* were found in high density exclusively in the cold and shaded water of springs in the sandstone massifs with or without vegetation and organic debris. Its presence was noted in Tagant, Assaba and Adrar region [19]. *Anopheles d’thali* larvae were present in all surface water of the Adrar region, even in those that are highly brackish. They were particularly abundant when the deposits contained filamentous green algae, on which eggs were abundant. In Hodh Elgharbi and Tagant, the larvae were found in fresh water sources with abundant aquatic vegetation and organic debris, and in residual spill of *Gueltas* (i.e. large rocky basin filled with water) without vegetation [20]. *Anopheles coustani* var. *ziemanni* larvae were collected in grassy swamps, ponds with *Pistia stratiofes* and streams with abundant vegetation. Water at breeding sites was sometimes very slightly brackish [20]. *Anopheles funestus* larvae were found in a large permanent pond with Pistia and slightly brackish water [20]. *Anopheles demeilloni* larvae were encountered in a stream from a permanent source with abundant vegetation and organic debris. *Anopheles gambiae* (*s.s.*) larvae were captured in a variety of breeding sites with sometimes slightly brackish water including residual puddles of ponds and rivers, ponds with or without silt and *Tamourts* (i.e. temporary pond of sub-desertic areas densely shaded by huge Acacia, streams near their origin), *Oglas* (i.e. shallow hole to reach ground water in the beds of temporary *wadis*), overflow areas of the Senegal River constituting flooded meadows, and a pond banco and grassy rivers. In Adrar and Trarza, larvae of *An. pharoensis* were caught in various mosquito development sites generally with clear or muddy and sometimes slightly brackish water characterized by the presence of aquatic vegetation such as ponds, *Tamourts*, residual pools, puddles and backwaters, rutted track, flooded meadows on the Senegal River and *Gueltas* in the Adrar region. Breeding sites where larvae of *An. pretoriensis* were collected in Hodh Elgharbi consisted of the affluent of permanent sources, with abundant vegetation and much organic debris. Larvae of *An. squamosus* were found in grassy flats of cool or warm water, often muddy, sometimes slightly brackish such as swamps, *Tamourts*, flooded meadows and streams. In Adrar, the breeding site was a large *Guelta*.

In Nouakchott, where *An. arabiensis* seems to be the sole *Anopheles* species thriving in this arid and human-made environment, water bodies consisting of water discharged from standpipes and household drinking water tanks served as *An. arabiensis* larval habitats [50, 51]. Using multivariate regression analyses, it was further shown that salinity up to 0.1 g/l and shaded habitats were protective factors against high larvae density in breeding sites and that pH up to 7.61 was a risk factor for high larvae density in these breeding sites [50].

Studies on the insecticide susceptibility of anopheline mosquitoes in Mauritania are limited. In 2005, Ba [44] tested the susceptibility to 0.75% permethrin and 0.05% deltamethrin, of *An. gambiae* (*s.l.*) and *An. pharoensis* from Boghé (Brakna), Rosso (Trarza), Sélibaby (Guidimagha) and Aioun (Hodh Elgharbi). For permethrin, *An. gambiae* (*s.l.*) specimens from all sites were sensitive. At Rosso and Boghé, there was 100% mortality among *An. pharoensis* females tested. For deltamethrin, only *An. pharoensis* specimens from Rosso were tested. The applied concentration resulted in 100% mortality.

The genetic profile of insecticide resistance was recently assessed in *An. gambiae* (*s.l.*) populations in Hodh Elgharbi (Sahelian zone) and Nouakchott (Saharan zone) [47]. Analysis of pyrethroid knockdown resistance (*kdr*) gene polymorphism showed the predominance of wild-type *kdr* L1014 among *An. arabiensis* in the two regions. Both *kdr* point mutations (L1014S and L1014F) were found to co-exist in the mosquitoes examined. However, the most important finding in this study was the presence of a high proportion of the east African mutation (L1014S) among *An. arabiensis* captured in Nouakchott. This mutation which was initially observed only in East Africa [52], now appears to be invading West Africa [53]. None of the assessed mosquitoes showed mutations in the acetylcholinesterase resistance (*ace-1*) gene.

### *Aedes* spp.

Fourteen *Aedes* species were described in Mauritania (Table 1). The presence of *Aedes* spp. was first documented in the late 1960s with the reports of *Ae. scatophagoides*, *Ae. metallicus* and *Ae. luteocephalus* in Kaedi (Gorgol) in the Senegal River valley, *Ae. vittatus*, *Ae. ochraceus* and *Ae. aegypti* in the southernmost regions of Gorgol, Assaba, Guidimagha and *Ae. hirsutus* in the southeastern regions of Hodh Elgharbi and Hodh Charghi [21, 35, 54, 55]. *Aedes vexans*, the chief enzootic vector subspecies of RVF, was collected from Trarza, Brakna, Assaba, Adrar and Hodh Elgharbi regions and Senegal using four different methods: CDC light traps, animal baited traps, landing catches on human baits and aspiration inside human dwellings [8, 9, 23, 44, 56]. Specimens of *Ae. vexans* and *Ae. sudanensis* were also captured in Tagant and Hodh Elgharbi regions, following the RVF outbreaks of 1998–1999 and 2012 [8, 23]. Entomological surveys carried out in Senegal River valley, few years after the first documented RVF outbreak that occurred in 1987 in southern Mauritania, suggested that *Ae. vexans* and probably *Ae. ochraceus* may be involved in the transmission of this epizootic disease since ten and three RVF virus strains were isolated from these two species during the survey, respectively [3]. Recently, the presence of *Ae. aegypti aegypti* and *Ae. caspius* was reported for the first time in Nouakchott [51]. DF outbreaks occurred in 2015 in the city (Ministry of Health, unpublished data) and specimens of *Ae. aegypti* have been regularly captured since that time (Mint Lekweiry, pers. comm.).

*Aedes aegypti aegypti*, *Ae. vittatus* and *Ae. caspius* immature stages were isolated from diverse habitats including abandoned containers, big canaries of drinking water, poultry water trough, household drinking water tanks, rock holes and rain puddles, respectively [35, 51, 54]. Furthermore, it has been shown that *Ae. aegypti* development sites in southern Mauritania were closely related to the habits of different ethnic groups regarding the mode of water storage. For instance, canaries of drinking water used by the Soninke people were the main larval development sites of the species in Guidimigha and part of Gorgol regions [35]. No data on insecticide resistance in this species is currently available for Mauritania.

### *Culex* spp.

A total of 18 *Culex* species were described in Mauritania (Table 1). These are *Cx. antennatus*, *Cx. decens*, *Cx. neavei*, *Cx. perfuscus*, *Cx. poicilipes*, *Cx. quinquefasciatus*, *Cx. perexiguus*, *Cx. tigripes*, *Cx. ethiopicus*, C*x. simpsoni*, *Cx. pipiens*, *Cx. fatigans*, *Cx. univittatus*, *Cx. duttoni*, *Cx. grahami*, *Cx. annulioris*, *Cx. nebulosus* and Cx. *tritaeniorhynchus* [8, 9, 21, 23, 35, 41]. Apart from the malariometric survey of Hamon et al*.* [21] in which ten *Culex* species were identified in different regions of Mauritania and that of Pichon and Ouedraogo [35] exploring potential conditions for the emergence of YF epidemics, where 16 *Culex* species have been described, all entomological investigations reporting the presence of *Culex* species were closely related to the epidemics of RVF in Mauritania. Indeed, during their survey of 1998, following the RVF outbreak in Hodh Elgharbi region (south-eastern Mauritania), Nabeth et al*.* [41] using CDC light traps with CO_2_ set nearby temporary ground pools and CDC light traps without CO_2_ set in sheepfolds or cowsheds, captured *Cx. antennatus*, *Cx. decens*, *Cx. neavei*, *Cx. perfuscus*, *Cx. poicilipes* and *Cx. quinquefasciatus* but they were not able to isolate RVF virus among captured mosquitoes. However, entomological investigations conducted in 1998 and 1999 in Hodh Elgharbi where the same RVF outbreak occurred and the Tagant central region, Diallo et al. [8] captured *Cx. poicilipes* specimens naturally infected with RVF virus. During the following RVF outbreaks in 2003 which affected Trarza, Brakna and Assaba southern regions of Mauritania and in 2012 which occurred in the northern Adrar region, *Cx. poicilipes* together with *Cx. antennatus* were found positive with RVF viruses [9, 12]. *Culex poicilipes* was already incriminated as RVF vector in Senegal during an entomological survey undertaken to assess the extent of virus circulation in this country following the re-emergence of the RVF virus in Hodh Elgharbi region in 1998 [57].

### Other *Culicidae*

Two *Mansonia* species, namely *Ma. uniformis* and *Ma. africana*, were reported to occur in Mauritania (Table 1). *Mansonia africana* was first collected in Gorgol by Hamon et al. [21]. Its presence was recently confirmed by Faye et al. [9], but these authors prospected 11 localities in Trarza, Brakna and Assaba regions without stating in which of them the specimens of *Mansonia* were collected. *Mansonia uniformis* was also collected either before 1970 or more recently either in the Saharan (Adrar and Tagant) or the Sahelian (Hodh Charghi, Trarza and Gorgol) regions of the country [9, 21, 23, 35]. Although females of both species were identified among biting mosquito populations in Mauritania, their medical importance has not yet been elucidated.

## Discussion

This report updates available knowledge on mosquito biodiversity in Mauritania through an extensive review of entomological findings gathered in the country from 1948 to 2016. Most of the reviewed literature dealt with the inventory, distribution and ecology of the mosquito fauna focusing on those of medical importance, particularly anopheline mosquitoes. While research carried out before 1970 was limited and was often part of the malaria pre-elimination programme at that time, entomological research gained momentum at the beginning of the twentieth century after the repeated outbreaks of RVF of 1987 and 1998 in the south and southeastern regions of Mauritania where the disease became endemic since that time [10].

Mosquito-transmitted diseases including malaria, RVF and DF have been reported, and for malaria, confirmed to occur in Mauritania. Fever outbreaks that occurred in 2014 and 2015 in Nouakchott were initially suspected to be malaria infections, but diagnosis using the commercial SD Bioline Dengue NS1 Ag Rapid Test (Standard Diagnostics Inc., Gyeonggi-do, South Korea) confirmed the infection with DF virus (Ouldabdallahi Moukah, unpublished data). They were later officially recognized by the Mauritanian Health officials and notified to the WHO in 2015 together with the RVF outbreak that occurred in Brakna region.

Malaria is transmitted by species of the *An. gambiae* (*s.l.*) complex, represented mainly by *An. arabiensis.* Its vectorial capacity was recently demonstrated in Nouakchott and in the southern region of Assaba [22, 46, 47]. The presence of *An. arabiensis* in Mauritania was first reported by the works of Hamon et al. [20] who suggested that the specimens of *An. gambiae* (*s.l.*) captured during their survey were most probably *An. gambiae* (species B), later known as *An. arabiensis* [58], which generally occurs in more arid habitats than the other members of the complex. *Anopheles arabiensis* is one of the three most efficient malaria vectors in Africa, and its wide distribution in Mauritania agrees with the general distribution of the species in Africa [59] and particularly in the neighboring country of Senegal [60]. *Anopheles gambiae* (*s.s.*), *An. funestus* and four secondary malaria vectors (*An. coustani*, *An. ziemanni*, *An. pharoensis* and *An. wellcomei*) were also reported, but their role as malaria vectors in Mauritania has not been established so far.

*Culex poicilipes* and *Cx. antennatus* were incriminated in the repeated RVF outbreaks and *Aedes aegypti* was probably responsible of the recent DF outbreaks occurring in Nouakchott in 2014 and 2015. However, at present, a definite proof linking these mosquito species to RVF and DF is yet to be established.

Spatial and temporal distribution of the Culicidae fauna in Mauritania is heterogeneous. The southern Gorgol and Trarza, and central Tagant and Assaba regions of the country are the richest in species diversity with 18, 17, 17 and 14 species over the 49 scored, respectively. Mosquito biodiversity is much lower in the northernmost regions. However, there are no or few data on the mosquito biodiversity from the southeastern Hodh Charghi region and from most of the northern Saharan regions of the country. Because oases might serve as “hubs” for human as well as livestock migration throughout the desert, entomological surveillance should be regularly implemented in these areas to explore the risk for pathogen emergence.

According to Hamon et al. [20], the fauna of central and southern Mauritania is closely related to those of Senegal, southern Mali and northern Sudan, while the fauna of the northern Saharan region of Adrar is likely to have more affinity with the arid regions of Chad, Somalia, Arabian Peninsula and North Africa.

The heterogeneity in the spatial and temporal distribution of the Culicidae biodiversity in Mauritania observed in the reviewed literature, could be explained, at least partially, by the differences in the investigation periods (dry season versus wet season), their short time period and occasional nature (most site were visited only once during an investigation and always after an epidemic) and the method with which mosquitoes were collected (light traps, human baited trap, larval collection, PSC, etc.).

However, it could be assumed that at least for some species, events of extinction or introduction have probably occurred. For instance, while Hudleston [61] suggested that mosquitoes of the *An. gambiae* (*s.l.*) complex had a northern limit that passed through Boutilimit (17°32′N) situated in the Saharo-Sahelian zone, *An. arabiensis* has recently been reported further to the north, particularly in Nouakchott (18°32′N), the capital city [47] and Rachid (18°47′N) in the Tagant region [44]. Its presence in Nouakchott is probably the cause of the establishment of malaria transmission in this city which was considered until the recent past as malaria-free [14, 15, 47]. Conversely, *An. melas* and *An. gambiae* (*s.s.*) have shrunk their distribution areas compared to the 1960s. Indeed, after the construction of the anti-salt Diama dam in 1986, on the Senegal river, *An. melas* populations previously recorded in the localities of Richard Toll (15°41′W) and Tounguen (15°46′W) in Senegal and Mauritania, respectively [20, 36] have moved westward to Saint-Louis (16°28′W) near the Atlantic coast, where saltwater breeding-places are present [38]. *Anopheles gambiae* (*s.s.*) has probably experienced a similar event but due, this time, to the establishment of extreme arid conditions in central Mauritania after the 1970–1990s drought.

The recent discovery of *Ae. aegypti* in Nouakchott [51] probably resulted from the expansion of more southern, contemporaneous populations thriving in sub-Saharan Africa. However, due to their limited sampling, these authors were not able to rule out direct invasion from Asia, through passive dispersal and human-assisted egg transportation by plane and ship owing to the homology of DNA sequence of the tested specimens with the published *Ae. aegypti* accessions from Vietnam and Thailand.

It has been reported that mosquito colonization/extinction process, population dynamics and vector capacity in a given area depend on several factors such as availability and type of larval development sites, climate and environmental changes, human population density, increased human travel and goods transport, and reduction of resources in the life-cycle of mosquitoes by interventions [62]. In Mauritania, climate and environmental changes, population and livestock movements, development of hydroagricultural projects, improvement of land transport infrastructures, and unplanned urbanization are probably the most relevant factors in this context. The degradation of the climatic conditions, caused by prolonged droughts during 1970–1990s in the Sahel resulted in a latitudinal shift of 100 mm isohyets for 100 km to the south between the periods 1947–1969 and 1970–1985. In Mauritania, isohyet 100 mm is even down by more than 200 km reaching Nouakchott region [63]. Desertification phenomenon then progressed southwards limiting the number and the longevity of aquatic habitats which probably affected the biodiversity and distribution of the Culicidae.

However, epidemics of vector-borne diseases, even in the northern Saharan parts of the country, remain a threat to public health particularly after natural disasters such as flooding caused by heavy rainfall. For instance, RVF outbreaks in 1998 and 2010 in the Hodh Elgharbi (Saharo-Sahelian) and the Adrar (Saharan) regions occurring after an exceptionally heavy rainfall resulted in highly favorable conditions for colonization and subsequent proliferation of competent vectors such as *Ae. vexans*, *Cx. poicilipes* and *Cx. antennatus* [10, 23].

Moreover, intense human and livestock movements occur annually in Mauritania. Human population movements are mostly seasonal and occur mainly between Nouakchott and the established malaria endemic zones. This population flux partly related to school vacation from July to the beginning of October had probably contributed to the introduction of infectious diseases, particularly malaria, from the southern endemic regions to the northern non-endemic regions and possibly contributed to the spread of *An. arabiensis* into the capital city [64]. The role of human population movements in the spread of communicable diseases such as malaria has long been recognized [65].

Livestock movements also occur throughout the year within Mauritania and from Mauritania to the neighboring Sahelian countries of Senegal and Mali. Cattles, small ruminants and camels are moved every year for grazing or sale, favoring virus circulation by introducing viraemic animals from an infected area to a receptive one as it has been suggested during the RVF outbreaks in Hodh Elgharbi and Adrar regions [10, 41].

Furthermore, the construction in 1986 of the Diama anti-salt Dam across the Senegal River coincided with the first RVF outbreak that occurred in 1987 in southern Mauritania and may have been its main contributing cause [7]. Indeed, Fontenille et al. [66] proposed that flooding of the river bank of the Senegal River in 1987, following construction of the dam, resulted in the increase of both mosquito and livestock densities and was probably the cause of this epizootic. RVF virus was isolated, 6 years later, from *Ae. vexans* and *Ae. ochraceus* mosquitoes and from one healthy sheep, in Barkedji area, located in the Sahelian Ferlo region of Senegal [3, 56] and in several localities in Senegal and Mauritania simultaneously following the RVF outbreak of 1998 in Hodh Elgharbi [57].

Unplanned urbanization due to rapid demographic growth characterized by poor housing, lack of sanitation, development of urban agriculture and inadequate surface water drainage is often cited as one of the major factors that influence the epidemiology of malaria and that of other emerging diseases, such as DF, in Nouakchott [50, 51]. This rapid urbanization creates new larval habitats and maintains the presence of both *An. arabiensis* and *Ae. aegypti* in the city.

To increase knowledge on the diversity and bionomics of mosquitoes in Mauritania, particularly those of medical importance, it is important to extend entomological surveys to the unexplored southeastern region of Hodh Charghi which is the most populated region in the country with 412,939 (12.2%) inhabitants in 2013 [25]. The region is also characterized by the presence of several permanent and semi-permanent wetlands locally known as *Tamourts*.

There is also a need to introduce more efficient and accurate techniques to collect and identify mosquito fauna. In this context, the establishment of an identification guide for larvae and adult female of common mosquitoes in Mauritania based on morphological criteria and/or molecular biology for species that are closely related such as members of the *An. gambiae* (*s.l.*) complex is an urgent task. This effort should be sustained by the development of skills and capacity building in medical entomology where the number of researchers at present does not meet the needs of a vast country like Mauritania.

As Mauritania moves towards malaria elimination by 2025, it will be necessary to continuously update the database of vector research and control by conducting entomological surveillance and evaluation, as much of the historical literature generated may not hold true at present and in the future, as environmental landscapes continue to change. Within the context of malaria elimination, the value of understanding the distribution and bionomics of *An. arabiensis* in Mauritania and its ability to sustain malaria transmission cannot be understated.

## Conclusions

The present review highlights that knowledge about Mauritanian mosquito fauna is closely related to human health rather than the result of systematic entomological surveillance. The Culicidae fauna of Mauritania comprises 17 *Anopheles* spp., 14 *Aedes* spp., 18 *Culex* spp. and two *Mansonia* spp. present in all ecological zones of Mauritania and including the major mosquito vectors of malaria, RVF and DF. Although this study represents important insights into the mosquito diversity of Mauritania, 51 species reported in this review are unlikely to be a complete inventory of the Mauritanian mosquito fauna probably because of the limited number of mosquito sampling methods and the lack of knowledge on biodiversity in southeastern and northern regions. A nationwide entomological field survey is necessary to update the list of mosquito species and establish an identification guide for both adult females and larval stages particularly for those of medical relevance.

## Abbreviations

(*s.l*.): *sensu lato*
(*s.s*.): *sensu stricto*
Ace-1: acetylcholinesterase resistance gene
CDC: Centers for Disease Control
DF: dengue fever
ELISA: enzyme-linked immunosorbent assay
*kdr*: knockdown resistance
PSC: pyrethrum space-spray catch
RVF: Rift Valley fever
WHO: World Health Organization
YF: yellow fever
